# *Ustilaginoidea virens* Nuclear Effector SCRE4 Suppresses Rice Immunity via Inhibiting Expression of a Positive Immune Regulator OsARF17

**DOI:** 10.3390/ijms231810527

**Published:** 2022-09-10

**Authors:** Shanshan Qiu, Anfei Fang, Xinhang Zheng, Shanzhi Wang, Jiyang Wang, Jing Fan, Zongtao Sun, Han Gao, Jiyun Yang, Qingtao Zeng, Fuhao Cui, Wen-Ming Wang, Jianping Chen, Wenxian Sun

**Affiliations:** 1 Department of Plant Pathology, The Ministry of Agriculture Key Laboratory of Pest Monitoring and Green Management, and Joint Laboratory for International Cooperation in Crop Molecular Breeding, Ministry of Education, China Agricultural University, Beijing 100193, China; 2 Rice Research Institute, Sichuan Agricultural University, Chengdu 611130, China; 3 State Key Laboratory for Quality and Safety of Agro-Products, Key Laboratory of Biotechnology in Plant Protection of Ministry of Agriculture of China and Zhejiang Province, Institute of Plant Virology, Ningbo University, Ningbo 315211, China; 4 College of Plant Protection, Jilin Agricultural University, Changchun 130118, China

**Keywords:** *Ustilaginoidea virens*, secreted cysteine-rich effector 4, rice false smut, auxin response factor 17, transcription inhibition

## Abstract

Rice false smut caused by the biotrophic fungal pathogen *Ustilaginoidea virens* has become one of the most important diseases in rice. The large effector repertory in *U. virens* plays a crucial role in virulence. However, current knowledge of molecular mechanisms how *U. virens* effectors target rice immune signaling to promote infection is very limited. In this study, we identified and characterized an essential virulence effector, SCRE4 (Secreted Cysteine-Rich Effector 4), in *U. virens*. SCRE4 was confirmed as a secreted nuclear effector through yeast secretion, translocation assays and protein subcellular localization, as well as up-regulation during infection. The *SCRE4* gene deletion attenuated the virulence of *U. virens* to rice. Consistently, ectopic expression of SCRE4 in rice inhibited chitin-triggered immunity and enhanced susceptibility to false smut, substantiating that SCRE4 is an essential virulence factor. Furthermore, SCRE4 transcriptionally suppressed the expression of *OsARF17*, an auxin response factor in rice, which positively regulates rice immune responses and resistance against *U. virens.* Additionally, the immunosuppressive capacity of SCRE4 depended on its nuclear localization. Therefore, we uncovered a virulence strategy in *U. virens* that transcriptionally suppresses the expression of the immune positive modulator OsARF17 through nucleus-localized effector SCRE4 to facilitate infection.

## 1. Introduction

Rice false smut caused by *Ustilaginoidea virens* is becoming one of the most important rice diseases worldwide because the disease not only causes severe yield losses but also greatly deteriorates grain quality due to mycotoxin contamination [1,2,3,4]. *Ustilaginoidea virens* has a unique infection style, that is, colonizing floral organs without observable penetration. The primary infection sites for the pathogen are stamen filaments, which are indispensable for the formation of false smut balls [3,5].

Transcriptome analysis revealed that many genes related to flower development and grain filling in rice are differentially expressed in *U. virens*-infected panicles, indicating that the pathogen acquires nutrient supply through hijacking the grain filling system for false smut ball development [6]. Among those genes, *OsARF17* (Os06g677800-01), encoding a family member of auxin response factors (ARFs), is transcriptionally suppressed during *U. virens* infection [6]. OsARF17, a key component of auxin signaling, regulates flag leaf inclination [7,8,9]. AtARF8 in *Arabidopsis*, a paralog of OsARF17, plays an important role in flower development and opening time [10,11]. Recently, OsARF17 has been identified to positively regulate plant resistance against multiple types of viruses. As a counter-defense mechanism, the SP8 protein of Southern rice black-streaked dwarf virus (SRBSDV) and P2 of rice stripe virus (RSV) interact with OsARF17 in different modes and suppress its transcriptional activation and DNA binding activity to interfere with auxin signaling [12]. Interestingly, auxin signaling in plants promotes infection of the hemibiotrophic fungal pathogen *Magnaporthe oryzae* and bacterial pathogens, including *Pseudomonas* spp., *Agrobacterium tumefaciens* and *Pantoea agglomerans* [13]. However, the role of OsARF17 in rice resistance against fungal infection is currently unknown.

Plants have evolved a sophisticated immune system to defend against pathogen invasion and colonization [14,15]. Pattern-triggered immunity (PTI), the first line of plant immunity, is activated by pattern recognition receptors (PRRs) through recognition of pathogen- (or microbe-) associated molecular patterns (PAMPs or MAMPs) [16]. On the other hand, the intracellular immune receptors specifically recognize certain effectors to initiate another layer of immunity, called effector-triggered immunity [17]. Filamentous pathogens secrete a large effector arsenal to disarm plant immunity through different molecular strategies [18,19,20,21]. Apoplastic effectors that are secreted into intercellular spaces of host cells are recognized by host plants to activate immunity or act as a virulence factor to suppress plant immunity [20,22]. For example, Ecp6 and Avr4 from *Cladosporium fulvum* and Slp1 from *Magnaporthe oryzae* bind chitin with high affinity through the LysM domains and thereby prevent chitin-triggered immunity [23,24,25,26,27]. On the other hand, pathogenic fungi can secrete numerous cytoplasmic effectors that function inside plant cells to play virulence functions or are recognized by R proteins to activate immune responses [20,22]. A glycine-serine-rich effector PstGSRE1 secreted by *Puccinia striiformis* targets the ROS-associated transcription factor TaLOL2, a positive regulator of wheat immunity, and hooks it out of the nucleus, therefore inhibiting ROS-mediated cell death to restrict the growth of the biotrophic pathogen [28]. Chorismate mutase (Cmu1) secreted by *Ustilago maydis* and unconventionally secreted isochorismatases in *Phytophthora sojae* and *Verticillium dahliae* impair salicylic acid (SA)-mediated resistance [29,30]. However, it is still largely mysterious how the majority of fungal effectors, particularly nuclear effectors, regulate plant immunity.

A total of 421 conventional and non-conventional effectors, including numerous nucleus-localized effectors, were predicted in *U. virens* genome [31,32]. The large effector arsenal promotes the infection and colonization of *U. virens*. Multiple secreted proteins and effectors, including SCRE1, SCRE2/UV_1261, SGP1 and SCRE6, were demonstrated to be essential for the full virulence of *U. virens* [33,34,35,36,37]. SCRE1 suppresses non-host hypersensitive response in *N. benthamiana* and host immunity through a small peptide region [35]. SCRE6 functions as a novel tyrosine phosphatase that dephosphorylates and stabilizes the negative immune regulator OsMPK6 [37]. A secreted chitin-binding protein UvCBP1 outcompetes chitin receptor OsCEBiP for chitin binding, thus inhibiting chitin-triggered immunity [38]. The effector UvSec117 recruits a negative immune regulator OsHDA701 into the nucleus and enhances OsHDA701-modulated deacetylation to interfere with histone H3K9 acetylation, thereby disrupting host immunity [39]. However, knowledge of molecular mechanisms how *U. virens* nuclear effectors suppress rice immunity is very limited.

The putative secreted cysteine-rich effector 4 (SCRE4) encoded by *UV8b_ 07665* in *U. virens* is a highly conserved protein in many pathogenic fungal species. In this study, we identify and characterize that SCRE4 is essential for *U. virens* virulence to rice. SCRE4 is secreted through the biotrophic interfacial complex (BIC) and is then translocated into the host nucleus during infection when the effector is heterologously expressed in *M. oryzae*. Furthermore, we show that SCRE4 suppresses the transcriptional expression of *OsARF17*, which plays a positive role in resistance to *U. virens*. Collectively, we identify a novel virulence strategy of the nucleus-localized effector SCRE4 to inhibit the expression of a positive immune regulator in rice.

## 2. Results

### 2.1. SCRE4 Is a Conserved Secreted Protein in U. virens

**Figure 1:**
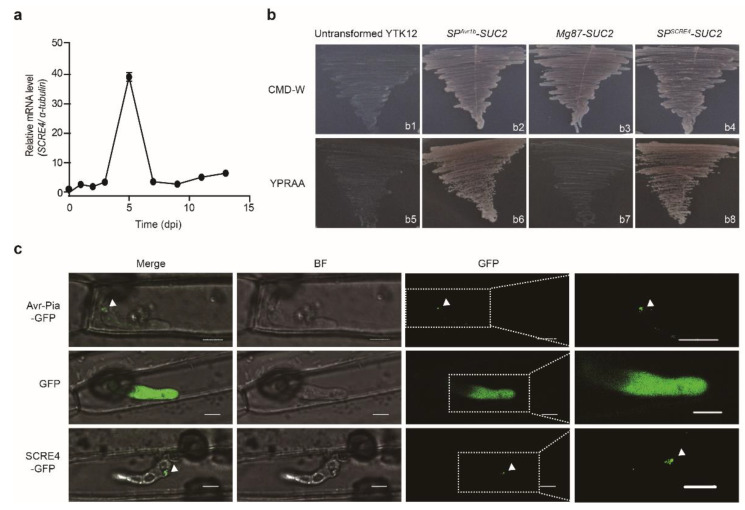
Identification of the secreted effector SCRE4 in *U.* *virens*. (**a**) Expression pattern of *SCRE4* during *U. virens* infection. The expression of *SCRE4* was detected in the inoculated panicles via quantitative RT-PCR at the indicated time points after the susceptible rice variety LYP9 was inoculated with *U. virens*. The *α-tubulin* gene was used as an internal reference. The representative data from three independent experiments are presented as mean ± standard error (SE) (*n* = 3). (**b**) Functionality of the putative signal peptide of SCRE4 confirmed by the yeast secretion system. b1–4: untransformed YTK12, *SP^Avr1b^-SUC2-*, *Mg87-SUC2-* and *SP^SCRE4^-SUC2*-transformed YTK12 strains were cultured on CMD-W medium, respectively; b5–8: the above-mentioned strains were cultured on YPRAA medium with raffinose as sole carbon source, respectively. *SP^Avr1b^-SUC2*, the signal peptide-encoding sequence of *P. sojae Avr1b* in fusion with the truncated *SUC2* gene. *Mg87-SUC2*, the N-terminal peptide-encoding sequence of non-secreted Mg87 in *M. oryzae* in fusion with the truncated *SUC2* gene. *SP^SCRE4^-SUC2*, the putative signal peptide-encoding sequence of *SCRE4* fused to the truncated *SUC2* gene. (**c**) Green fluorescence from SCRE4*-*GFP and Avr*-*Pia*-*GFP (a positive control) observed in BICs during *M. oryzae* infection. Leaf sheaths were inoculated with *M. oryzae* strains transformed with pYF11-*ProRP27:SCRE4-GFP*, pYF11-*ProRP27:Avr-Pia-GFP* or pYF11-*ProRP27:GFP*. The images were captured by confocal microscopy at 30 h after inoculation. BICs are indicated by white triangles. GFP, green fluorescent protein; BF, bright field; Merge, the overlay of GFP and BF images; Images on the right are enlarged from the blocks in broken squares in the GFP panels. Scale bar = 5 μm.

The putative secreted cysteine-rich effector 4 (SCRE4) encoded by *UV8b_ 07665* was predicted in *U. virens* with a putative signal peptide (SP). Furthermore, SCRE4 was predicted to contain a monopartite nuclear localization signal through cNLS Mapper and a conserved ‘RHG’ motif that is characteristic of the histidine phosphatase superfamily members (Figure S1a). BLAST searches showed that SCRE4 homologs are present in many phytopathogenic ascomycetes, including *Fusarium vanettenii*, *Aspergillus fumigatus*, *Neonectria ditissima*, *Peltaster fructicola* and *Dothistroma septosporum* and in multiple entomopathogenic fungi such as *Moelleriella libera*, *Metarhizium album*, *M. anisopliae*, *Akanthomyces lecanii* and *Beauveria bassiana*. The constructed phylogenetic tree of SCRE4 homologs from these fungal species substantiated the conservation of SCRE4 (Figure S1b). However, some homologs lack signal peptide and nuclear localization signals, implying that homologs may play other roles in the physiological process. In addition, the alignment of *SCRE4* coding sequences (CDSs) from 31 *U. virens* isolates revealed no nucleotide polymorphism, indicating that *SCRE4* is highly conserved in *U. virens* (Figure S1c). The conservation of SCRE4 implies its necessity in fungal virulence and pathogenicity. Furthermore, the expression pattern of *SCRE4* was also examined during *U. virens* infection. Quantitative RT-PCR (RT-qPCR) showed that the expression of *SCRE4* was highly up-regulated during the early stage of infection and was subsequently reduced at the late infection stage (Figure 1a), suggesting that SCRE4 plays an important role in *U. virens* infection.

First, we performed the yeast secretion assay to validate the functionality of the SCRE4 signal peptide [40,41]. The putative signal peptide-encoding sequence of *SCRE4* fused in frame with truncated *SUC2* gene, which encodes invertase without its own signal peptide. The construct was transformed into YTK12 strain. All transformed YTK12 strains were able to grow normally in the complete minimal medium lacking tryptophan (CMD-W medium), and the *SP^SCRE4^-SUC2*-transformed YTK12 strain grew well on the YPRAA medium with raffinose as the sole carbon source, indicating that the signal peptide of SCRE4 can guide the secretion of invertase into the medium to degrade raffinose. The constructs *SP^Avr1b^-SUC2* and *Mg87-SUC2* were transformed into YTK12 yeast cells as positive and negative controls, respectively ([33], Figure 1b).

Next, we determined whether SCRE4 is translocated into plant cells during infection using the translocation system of *M. oryzae* [35,42]. The *M. oryzae* transformants expressing SCRE4-GFP were inoculated into detached rice sheaths. At 30 h after inoculation, strong green fluorescence was observed in the BICs, a plant-derived membrane-rich structure, indicating that SCRE4 expressed in *M. oryzae* is secreted into rice cells through BICs during infection. *M. oryzae* strains expressing Avr-Pia-GFP and GFP were inoculated into rice sheaths as positive and negative controls, respectively. Avr-Pia-GFP was observed to be accumulated in BICs, whereas GFP was observed throughout the infected hyphae (Figure 1c). These results illustrated that ectopically expressed SCRE4 in *M. oryzae* was secreted and translocated into plant cells. Therefore, these results indicate that SCRE4 is an effector protein that is translocated into plant cells during infection.

### 2.2. SCRE4 Is An Essential Virulent Factor

**Figure 2:**
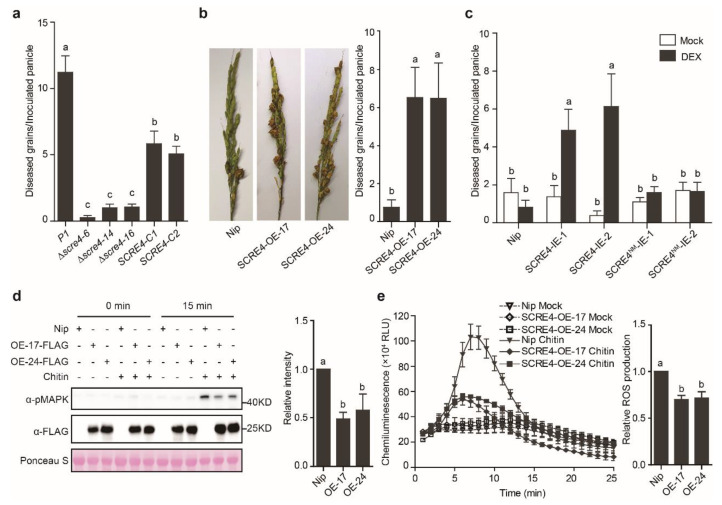
SCRE4 is an essential virulent factor in *U. virens*. (**a**) The average number of diseased grains per inoculated panicle. The *U. virens* wild−type (P1), Δ*scre4* knockout mutant and complemented strains (*SCRE4−C1* and *C2*) were injection-inoculated into young panicles of the susceptible rice cultivar LYP9 (*n* = 14, 15, 15, 15, 12, 13). (**b**) Disease symptoms and diseased grains on rice panicles of the wild−type (Nip), SCRE4−OE−17 and −24 lines after inoculation of the *U. virens* isolate JS60−2 (*n* = 12, 13, 10). Left images showed disease symptoms on the representative rice panicles after *U. virens* inoculation. (**c**) Diseased grains on *U. virens*−inoculated panicles of the wild−type (Nip), SCRE4−IE and SCRE4^NM^−IE lines. The wild−type and different transgenic lines were treated with 30 µM DEX and mock solution followed by injection inoculation with JS60−2 (*n* = 12, 11, 8, 8, 8, 8, 10, 10, 10, 9). In panels (**a**–**c**), diseased grains were counted at 4 weeks after inoculation. The representative data from three independent experiments are shown as mean ± SE. Different letters (**a**–**c**) indicate significant differences in the average number of diseased grains on rice panicles after inoculation of different strains (**a**), on the panicles of the wild−type and SCRE4−OE transgenic lines (**b**), and on the SCRE4−IE panicles after DEX and mock treatments (**c**) (*p* < 0.05, Duncan’s multiple range test). (**d**) Chitin−triggered MAPK activation in the wild−type and *SCRE4*−overexpressing transgenic lines. Total protein loading is indicated by Ponceau S staining. Right panel, the chitin−activated MAPK phosphorylation levels normalized to total proteins from three independent experiments are shown as mean ± SE (*n* = 3). Band intensity was determined by densitometry using ImageJ. Different letters (**a** vs. **b**) indicate a significant difference in the MAPK phosphorylation level (*p* < 0.05, Duncan’s multiple range test). (**e**) Chitin−triggered ROS burst in the wild−type and *SCRE4*−overexpressing transgenic lines. Right panel, relative ROS production in Nipponbare and *SCRE4*−overexpressing transgenic lines induced by chitin from three independent experiments are shown as mean ± SE. The total ROS levels were quantified by measurement of peak areas under the curve of ROS burst using GraphPad Prism5. Different letters (**a** vs. **b**) indicate a significant difference in the relative ROS level in the wild−type (Nip) and *SCRE4*−overexpressing transgenic lines (*p* < 0.05, Duncan’s multiple range test).

In order to determine the role of *SCRE4* during *U. virens* infection, the *SCRE4*-knockout mutants were generated using the CRISPR/Cas9 system and were then confirmed via PCR and Southern blot analyses (Figure S2a). The complemented strains were constructed by introducing the full-length *SCRE4* gene with the native promoter into the mutant strains, and the expression of SCRE4-FLAG in complemented strains was confirmed via immunoblotting (Figure S2c). The wild-type, mutant and complemented strains were then injected into young panicles of the susceptible rice cultivar LYP9 before heading. The Δ*scre4–*6, Δ*scre4–*14 and Δ*scre4–*16 mutants generated significantly fewer false smut balls on rice panicles than the wild-type and complemented strains after inoculation. The virulence of the complemented strains was largely restored (Figure 2a and Figure S2b). These results indicate that *SCRE4* plays an essential role in *U. virens* virulence to rice.

Next, we generated the transgenic rice lines with constitutive or conditional expression of SCRE4 to determine the virulence functions of SCRE4. The homozygous transgenic lines with SCRE4-FLAG expression under the control of maize ubiquitin promoter (called SCRE4-OE-17 and SCRE4-OE-24 hereinafter) or dexamethasone (DEX)-inducible promoter (called SCRE4-IE-1 and SCRE4-IE-2 hereinafter) were identified via immunoblotting (Figure S2d,e). After inoculation with the virulent strain JS60-2, the SCRE4-OE-17 and -24 lines produced significantly more diseased grains on inoculated panicles than did the wild-type Nipponbare plants (Figure 2b). Similarly, diseased grains formed on inoculated panicles of the DEX-treated SCRE4-IE-1 and SCRE4-IE-2 lines were many more than those in the wild-type plants (Figure 2c and Figure S2f). In addition, chitin-triggered mitogen-activated protein kinase (MAPK) activation and oxidative burst were greatly attenuated in the SCRE4-OE-17 and SCRE4-OE-24 lines compared with the wild-type plants (Figure 2d,e). Altogether, these results indicate that SCRE4 inhibits rice immunity and enhances disease susceptibility to false smut.

### 2.3. SCRE4 Is Internalized into Plant Cells and Localized in Nucleus

**Figure 3:**
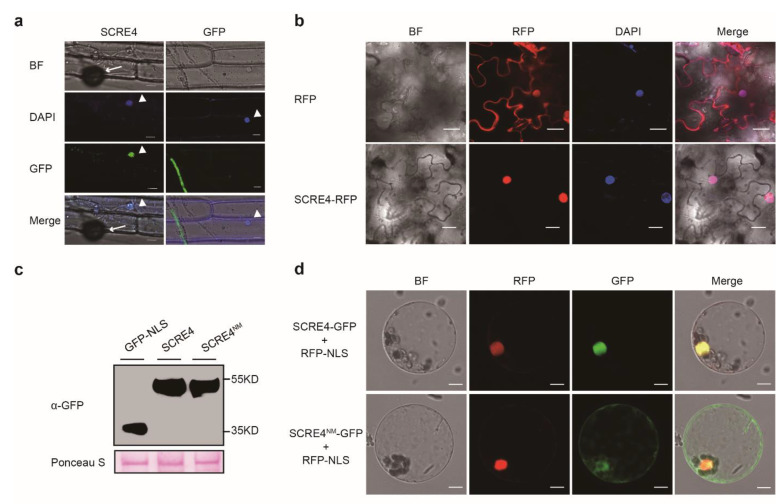
SCRE4 is internalized into plant cells during infection and is localized in nucleus. (**a**) Green fluorescence from SCRE4-GFP and GFP observed in rice cell nuclei during *M. oryzae* infection. The *M. oryzae* strains carrying pYF11-*ProRP27:SCRE4-GFP* and pYF11-*ProRP27:GFP* were inoculated onto detached rice sheaths. Green fluorescence was observed in epidermal cells of rice sheaths at 42 h after inoculation. BF, bright field; DAPI, the nuclei were stained with the dye DAPI; GFP, green fluorescent protein; Merge, the overlay of BF, GFP and DAPI images; Cell nuclei are indicated by white triangles, and appressoria are indicated by white arrows. Scale bar = 5 μm; (**b**) The subcellular localization of SCRE4-RFP and RFP expressed in *Nicotiana benthamiana* cells. SCRE4-RFP was transiently expressed in *N. benthamiana* leaves through *Agrobacterium*-mediated transient expression. The images were captured at 48 h after agro-infiltration under confocal microscopy. BF, bright field; RFP panels, red fluorescence from SCRE4-RFP; DAPI panels, the nuclei were stained with the dye DAPI; Merge, the overlay of GFP and DAPI images; Scale bar = 20 μm. (**c**) The expression of SCRE4-GFP and SCRE4^NM^ -GFP in rice protoplasts detected by immunoblotting with anti-GFP antibody (α-GFP). Protein loading is indicated by Ponceau S staining. (**d**) Subcellular localization of SCRE4-GFP and SCRE4^NM^-GFP expressed in rice protoplasts. RFP-NLS was transiently co-expressed with SCRE4-GFP or SCRE4^NM^-GFP in rice protoplasts. GFP and RFP signals were visualized under confocal microscope. RFP-NLS was used as a marker for nuclear localization. Scale bar = 7.5 μm.

SCRE4 was predicted to contain a monopartite nuclear localization signal (NLS) through cNLS Mapper (Figure S1a). In order to confirm whether SCRE4 was localized in the plant nuclei during infection, the *M. oryzae* strain carrying pYF11-*ProRP27:SCRE4-GFP* was inoculated onto detached rice sheaths. Fluorescence microscopy showed that green fluorescence was accumulated in rice cell nuclei at 42 h after inoculation, which was indicated by 2-(4-amidinophenyl)-6-indolecarbamidine (DAPI) staining (Figure 3a). In order to further determine the nuclear localization of SCRE4, SCRE4-RFP was transiently expressed in *N. benthamiana* leaves through *Agrobacterium*-mediated transient expression. Red fluorescence from SCRE4-RFP was only observed in the nuclei, which were indicated by DAPI staining (Figure 3b). Furthermore, green fluorescence from SCRE4-GFP and red fluorescence from RFP-NLS overlapped in nuclei when SCRE4-GFP and RFP-NLS were transiently expressed in rice protoplasts. By contrast, the mutant protein SCRE4^R73A/R75A^ (called SCRE4^NM^), in which the key Arg residues (Arg73 and Arg75) in the predicted NLS were both replaced with Ala, was predominantly observed in the cytosol when it was transiently expressed in rice protoplasts (Figure 3d). The expression of SCRE4-GFP and SCRE4^NM^-GFP was also detected by immunoblotting (Figure 3c). Collectively, these results indicate that SCRE4 is nucleus-localized, and the Arg residues in the predicted NLS are required for nuclear localization.

### 2.4. SCRE4 Transcriptionally Suppresses OsARF17 Expression

**Figure 4:**
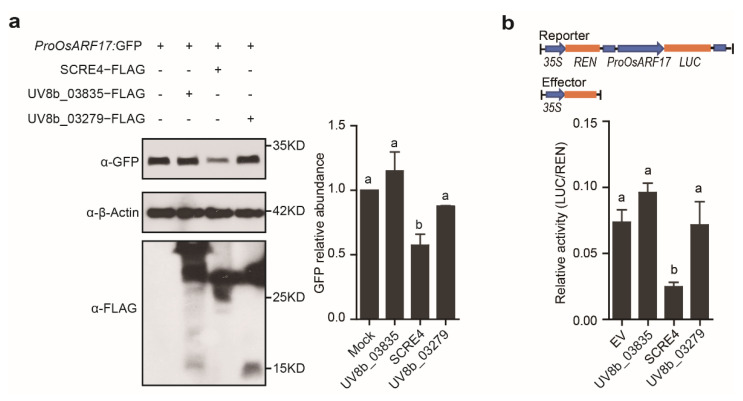
The transcriptional expression of *OsARF17* is suppressed by the *Ustilaginoidea virens* effector SCRE4. (**a**) The GFP expression level driven by the *OsARF17* promoter when it was individually co−expressed with SCRE4, UV8b_03835 and UV8b_03279 in rice protoplasts. The *ProOsARF17:GFP* construct was transfected alone or co−transfected with different putative effector gene constructs into rice protoplasts. The blots were detected via immunoblotting using anti−GFP, anti−β−Actin and anti−FLAG antibodies. The band intensity was quantified using ImageJ. Relative GFP abundance was normalized to the level of β−Actin. Right panel, data from three independent experiments are shown as mean ± SE. Different letters (**a** vs. **b**) indicate a significant difference in the GFP expression level (*p* < 0.05, Duncan’s multiple range test). (**b**) The dual−LUC assay to show the relative LUC/REN activity when the *ProOsARF17:LUC* construct was co−transformed with different putative effector gene constructs in *N. benthamiana*. Upper diagram shows the features of the constructs used in the dual−LUC assay. The relative LUC/REN activity was measured after LUC, and putative effectors were transiently co−expressed in *N. benthamiana* leaves. Different letters (**a** vs. **b**) indicate a significant difference in the relative activity (LUC/REN) when LUC was co−expressed with different proteins (*p* < 0.05, Duncan’s multiple range test).

*OsARF17* (Os06g677800-01), a putative flower development-related gene, is transcriptionally suppressed during *U. virens* infection [6]. We investigated whether any putative effectors in *U. virens* inhibit the expression of *OsARF17* through transient expression in rice protoplasts. Multiple putative effector gene constructs were individually transfected with *ProOsARF17:GFP* into rice protoplasts. Western blot analyses showed that GFP expression driven by the *OsARF17* promoter was significantly suppressed by SCRE4 co-expression but was not inhibited by other tested putative effectors, UV8b_03835 or UV8b_03279 (Figure 4a). In order to confirm whether SCRE4 inhibits transcriptional expression of *OsARF17*, we performed the dual luciferase (Dual-LUC) reporter assay in *N. benthamiana*. The firefly LUC reporter was expressed under the control of the *OsARF17* promoter, and Renilla luciferase (REN) was expressed under the *35S* promoter as an internal reference (Figure 4b). The results showed that the relative reporter activity (LUC/REN) was significantly inhibited by SCRE4 co-expression but was not by co-expression with UV8b_03835, UV8b_03279 or empty vector (Figure 4b). These data confirmed that SCRE4 transcriptionally inhibits the expression of *OsARF17*.

### 2.5. OsARF17 Positively Regulates Rice Defense against U. Virens

**Figure 5:**
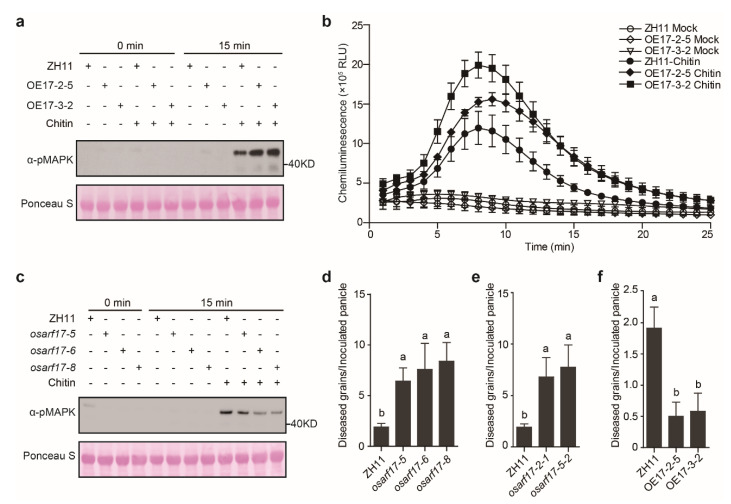
OsARF17 positively regulates rice defense against *U. virens*. (**a**) The chitin−induced MAPK phosphorylation levels in the wild−type and *OsARF17*−overexpressing lines. MAPK phosphorylation was detected by immunoblot analyses with anti−phospho−44/42 MAPK antibody (α−pMAPK). Protein loading is indicated by Ponceau S staining. (**b**) Chitin−triggered ROS burst in the wild−type and *OsARF17*−overexpressing lines. The leaves of the wild−type and transgenic lines OE17−2−5 and OE17−3−2 were treated with chitin (10 µg mL^−1^) or mock control. ROS burst was detected immediately after treatment within 25 min. (**c**) Chitin−induced MAPK activation detected in the wild−type and *osarf17* mutant lines. Chitin treatment and detection of MAPK phosphorylation were performed as described in (**a**). (**d**–**f**) The average number of diseased grains on inoculated panicles of the wild−type (ZH11) and different transgenic lines. The virulent *U. virens* isolate PJ52 was injection-inoculated into the wild−type and knockout lines *osarf17−5*, *osarf17−6* and *osarf17−8* (*n* = 12, 14, 10, 10) (**d**), the wild−type and mutant lines *osarf17−2−1* and *osarf17−5−2* generated with another sgRNA site (*n* = 12, 5, 8) (**e**), and the wild−type, *OsARF17*−overexpressing OE17−2−5 and OE17−3−2 lines (*n* = 12, 12, 12) (**f**). False smut balls were counted on the inoculated panicles 4 weeks after inoculation. The representative data from three independent experiments are shown as mean ± SE. Different letters (**a** vs. **b**) indicate a significant difference in the average number of diseased grains between the wild−type and different transgenic lines (*p* < 0.05, Duncan’s multiple range test).

In order to investigate whether OsARF17 positively regulates plant immunity, PAMP-induced ROS burst and MAPK phosphorylation were examined in the *OsARF17*-overexpressing and *osarf17* knockout lines after chitin treatment. Compared with the wild-type plants, the *OsARF17*-overexpressing lines OE17-2-5 and OE17-3-2 exhibited enhanced chitin-triggered MAPK phosphorylation and ROS burst (Figure 5a,b), whereas chitin-induced MAPK activation in the knockout lines *osarf17-5*, *osarf17-6* and *osarf17-8* were attenuated (Figure 5c). Furthermore, inoculation assays were performed by injection of the virulent strain PJ52 into rice panicles of the wild-type and different transgenic rice lines. The results showed that all the tested *osarf17* mutant lines generated with two sgRNA target sites produced significantly more diseased grains on the inoculated panicles, whereas the OE17-2-5 and OE17-3-2 lines generated many fewer false smut balls than did the wild-type plants (Figure 5d–f and Figure S3a–c). Altogether, these data indicate that OsARF17 plays an important role in rice resistance to false smut disease.

### 2.6. Immunosuppressive Ability of SCRE4 Is Dependent on Nuclear Localization

**Figure 6:**
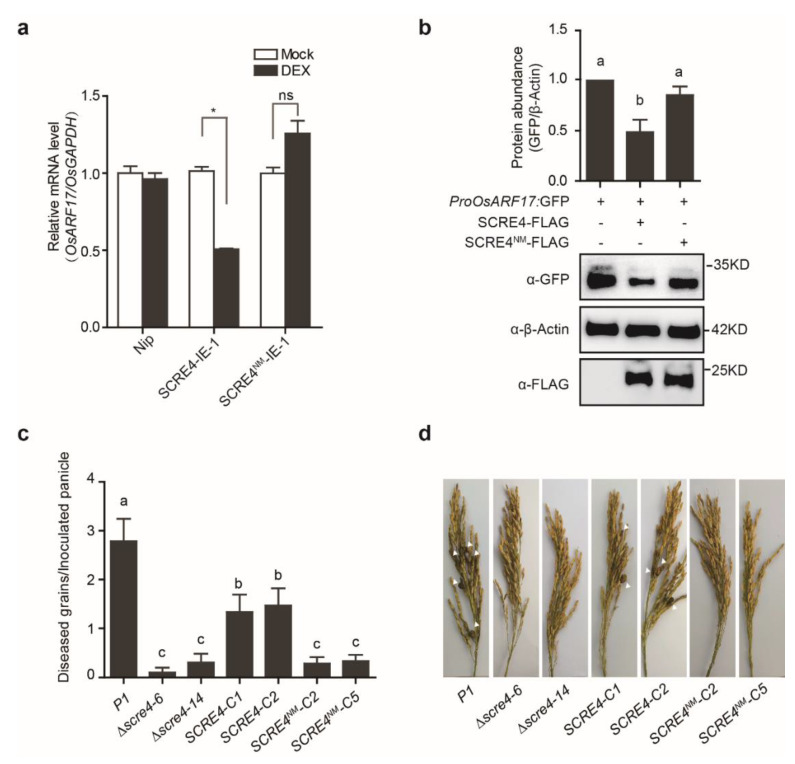
The ability of SCRE4 to suppress rice immunity is dependent on nuclear localization. (**a**) Expression of *OsARF17* in the wild−type, SCRE4−IE and SCRE4^NM^−IE transgenic lines after DEX and mock treatments. *OsGAPDH* expression was used as an internal reference. Asterisk indicates significant difference in the expression level of *OsARF17* in the SCRE4−IE−1 line between DEX and mock treatments (Student’s *t*-test, * *p* < 0.05). (**b**) The GFP expression level driven by the *OsARF17* promoter when the *ProOsARF17: GFP* construct was co-transfected with the *SCRE4* or *SCRE4^NM^* constructs into rice protoplasts. Total proteins were subject to Western blot analyses probed with anti−GFP, anti−β−Actin and anti−FLAG antibodies. Upper panel, the band intensity was quantified with Image J. Data from three independent assays are shown as mean ± SE (*n* = 4). Different letters ((**a**) vs. (**b**)) indicate a significant difference in relative GFP abundance between SCRE4− and SCRE4^NM^−expressing protoplasts (Duncan’s multiple range test, *p* < 0.05). (**c**) Diseased grains on rice panicles after inoculation of different *U. virens* strains. The *U. virens* wild−type (P1), Δ*scre4* knockout mutant and complemented strains (*SCRE4−C1* and *C2*, *SCRE4^NM^−C2* and *C5*) were injection-inoculated into young panicles of the susceptible rice cultivar LYP9 (*n* = 14, 10, 13, 15, 15, 14, 15). (**d**) Disease symptoms on the representative rice panicles after *U. virens* inoculation. The images were captured 4 weeks after rice panicles of LYP9 were inoculated by the wild−type (P1), different *scre4* knockout and complemented strains (*SCRE4−C1* and *C2*, *SCRE4^NM^−C2* and *C5*). False smut balls are indicated by white triangles.

In order to explore the role of SCRE4 nuclear localization in suppressing immune responses in rice, the SCRE4^NM^-IE-1 and SCRE4^NM^-IE-2 transgenic lines with DEX-induced expression of SCRE4^NM^-FLAG were generated and confirmed via immunoblotting (Figure S4a). By contrast, these SCRE4^NM^-IE transgenic lines exhibited no significant difference in the number of diseased grains on inoculated panicles after DEX and mock treatments (Figure 2c and Figure S2f). Furthermore, the transcript level of *OsARF17* was detected in the SCRE4-IE-1 transgenic line after DEX and mock treatments via RT-qPCR. The results showed that the expression of *OsARF17* was significantly reduced in the SCRE4-IE-1 transgenic line after DEX treatment compared with the mock control. By contrast, DEX-induced expression of SCRE4^NM^-FLAG in the SCRE4^NM^-IE-1 transgenic line did not significantly alter the expression of *OsARF17* (Figure 6a). Consistently, SCRE4 but not SCRE4^NM^ transiently expressed in rice protoplasts suppressed GFP expression driven by the promoter of *OsARF17* (Figure 6b). These data indicate that SCRE4 suppresses the transcript level of *OsARF17* and that the suppression ability is dependent on nuclear localization.

In order to further confirm the importance of SCRE4 nuclear localization in *U. virens* virulence, the *SCRE4^NM^-FLAG* driven by the native promoter was introduced into the *scre4* mutant, and the expression was confirmed via immunoblotting (Figure S4b). The wild-type, mutant and complemented strains with *SCRE4* or *SCRE4^NM^* were injected into young panicles of rice cultivar LYP9. Compared to the wild-type and complemented strains with the plasmid-borne *SCRE4* gene, the complemented strains with the plasmid-borne *SCRE4^NM^* construct generated significantly fewer false smut balls on rice panicles after inoculation, indicating that the virulence of the complemented strains with *SCRE4^NM^* was not restored (Figure 6c,d). These results indicate that SCRE4 inhibits rice immunity and enhances disease susceptibility to false smut and that its nuclear localization is important for immunosuppressive ability.

## 3. Discussion

*Ustilaginoidea virens* is an increasingly important fungal pathogen that specifically infects rice florets. The rice-*U. virens* interaction offers a unique pathosystem to understand the pathogenicity mechanisms of the flower-colonizing pathogen. In this study, we revealed that the nuclear effector SCRE4 plays an important role in *U. virens* virulence to rice. SCRE4 transcriptionally inhibits the expression of the immune positive regulator OsARF17, thereby suppressing plant resistance in rice.

In our previous study, we predicted SCRE4 encoded by *UV8b_ 07665* as an effector in *U. virens*, which has a putative signal peptide and inhibits hypersensitive responses in *N. benthamiana* ([31], *UV_6647*). Here, we demonstrated that the putative signal peptide of SCRE4 is functional in guiding the secretion of invertase (Figure 1b). It has been well established that bacterial effectors are injected into plant cells through the type III secretion system [43]. Presumably, many intracellular effectors in filamentous fungi are secreted into the extracellular spaces of host cells under the guidance of signal peptides before being translocated into plant cells [44]. *Ustilaginoidea virens* only infects rice floral organs before heading, as yet no method has been developed to study effector translocation in *U. virens*. Therefore, we exploited the rice-*M. oryzae* pathosystem and demonstrated that ectopically expressed SCRE4-GFP was accumulated in the BICs during *M. oryzae* infection, indicating that SCRE4-GFP is secreted into host cells via the BICs (Figure 1c). More convincingly, green fluorescence from SCRE4-GFP was observed in rice cell nuclei at the later stage of infection (Figure 3a), indicating that SCRE4-GFP in *M. oryzae* is secreted and translocated into plant cell nuclei. In addition, we showed that fluorescence protein-tagged SCRE4 was predominantly localized into the nuclei when it was transiently expressed in *N. benthamiana* cells and in rice protoplasts (Figure 3b–d). The conserved basic residues Arg73 and Arg75 in NLS are essential for nuclear localization (Figure S1a and Figure 3c,d) and transcription suppression activity in rice protoplasts and in transgenic lines (Figure 2c, Figure 6 and Figure S2f). These findings indicate that the immunosuppressive ability of SCRE4 is dependent on its nuclear localization.

Furthermore, we revealed the necessity of SCRE4 in *U. virens* virulence through a series of experiments. First, we illustrated that the virulence of the Δ*scre4* mutants was attenuated compared with the wild-type and complemented strains (Figure 2a and Figure S2b). Second, the *SCRE4*-overexpressing transgenic lines showed an evident suppression of chitin-triggered MAPK activation and ROS generation (Figure 2d,e). More convincingly, constitutive and induced expression of SCRE4 caused the transgenic rice lines to be more susceptible to *U. virens* JS60-2 (Figure 2b,c and Figure S2f). Together with the induced expression pattern of *SCRE4* during *U. virens* infection (Figure 1a), these results demonstrated that SCRE4 is an essential virulence factor in *U. virens*.

Multiple nuclear effectors from phytopathogenic fungi have been identified to target essential immune components through different mechanisms. For example, the effector CgEP1 from *Colletotrichum graminicola* targets host nuclei and is required for maize anthracnose development [45]. The nucleus-localized effector VdSCP41 in *V. dahliae* interacts with the *Arabidopsis* master immune regulators CBP60g and SARD1 and cotton GhCBP60b to inhibit the transcriptional expression of defense genes [46]. The two nuclear *M. oryzae* effectors MoHTR1/2 reprogram the expression of immunity-associated genes in rice [47]. Through multiple screenings, SCRE4, among the putative effectors that suppress hypersensitive responses in *N. benthamiana* [31], was identified to transcriptionally inhibit *OsARF17* expression in rice protoplasts (Figure 4a,b). Consistently, *OsARF17* is transcriptionally suppressed during *U. virens* infection [6]. Additionally, the transcript level of *OsARF17* was significantly reduced after conditional expression of SCRE4 in the transgenic lines (Figure 6a). The auxin response factor OsARF17 activates auxin signaling by modulating the transcription of auxin-regulated genes [9,12]. The role of host auxin signaling during pathogen infection depends on different types of pathogens and the nature of the host-pathogen interaction [13]. For example, some necrotrophic pathogens, such as *Alternaria* and *Rhizoctonia*, stimulate auxin signaling, resulting in the activation of defenses that inhibit pathogen replication and spread [48,49]. However, some biotrophic bacteria, such as *Pseudomonas* spp., *A*. *tumefaciens* and *P*. *agglomerans*, and the fungal pathogen *M. oryzae*, induce auxin signaling to promote pathogen infection [13,50]. An elegant study revealed that OsARF17 acts as a positive regulator in immunity against several plant viruses [12], indicating that the activation of auxin signaling causes an enhanced resistance to virus infection in plants. Our data demonstrated that OsARF17 positively regulates plant immune responses, including PAMP-induced ROS burst and MAPK activation, and thereby enhances resistance against rice false smut (Figure 5 and Figure S3). Next, it will be interesting to explore the molecular mechanisms of OsARF17 in regulating plant immunity against different plant pathogens. In addition, OsARF17 also plays a role in rice growth and development besides immunity regulation. Multiple studies revealed that OsARF17 affects the flag leaf angle in rice by regulating secondary cell wall biosynthesis of lamina joints [9,51] and tiller angle [52]. However, it is unclear how OsARF17 not only regulates plant growth and development but also modulates disease resistance in rice.

**Figure 7:**
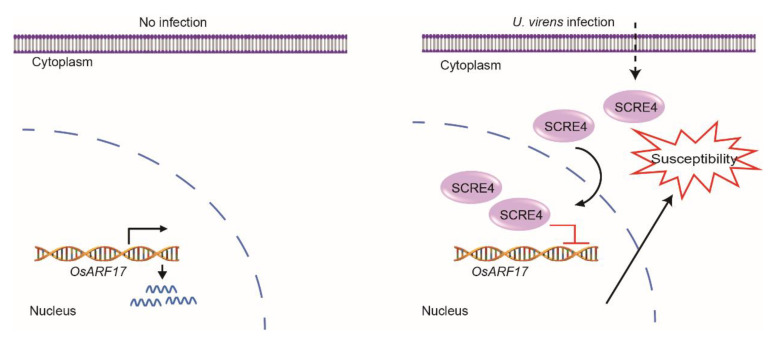
A working model of SCRE4 suppressing rice immunity. The auxin response factor OsARF17 functions as a positive regulator of PTI responses and positively regulates rice resistance to false smut. During *U. virens* infection, the essential virulence effector SCRE4 is secreted and translocated into rice nuclei. Through an unidentified mechanism, SCRE4 transcriptionally suppresses *OsARF17* expression in the nucleus and subsequently inhibits MAPK activation and ROS production. Therefore, immune responses in rice are disarmed, thus promoting *U. virens* infection.

Based on the above findings, we propose a working model for *U. virens* to suppress rice immunity through SCRE4 action (Figure 7). OsARF17 is a positive regulator of PTI responses and plant immunity against rice false smut. During *U. virens* infection, SCRE4 is secreted and translocated into rice cells. As a nucleus-localized effector, SCRE4 transcriptionally suppresses *OsARF17* expression in the nucleus and thereby inhibits MAPK activation and ROS production to promote *U. virens* infection. SCRE4 may directly bind the promoter of *OsARF17* as a transcriptional suppressor or indirectly interferes with other transcription factors to inhibit *OsARF17* expression. Therefore, it is an interesting topic to elucidate how SCRE4 inhibits *OsARF17* expression and rice immunity in the future.

## 4. Materials and Methods

### 4.1. Microbial Strains, Plant Materials and Growth Conditions

The *U. virens* isolates P1, JS60-2 and PJ52 were cultured in potato sucrose agar (PSA) medium (the filtrate of 200 g boiled potatoes, 20 g sucrose, 15 g agar per liter). The *Agrobacterium tumefaciens* GV3101 and EHA105 strains were cultured in Luria Bertani (LB) broth at 28 °C. The yeast strain YTK12 was cultured on a YPDA medium (1% yeast extract, 2% peptone, 2% glucose, 0.003% adenine hemisulfate, 2% agar). *Nicotiana benthamiana* plants were grown in a growth chamber under a 16/8-h day/night cycle at 25 °C. *Oryza sativa* subsp. *japonica* cv. Nipponbare (NIP) and Zhonghua11 (ZH11), and the derivative transgenic rice plants, were grown in the greenhouse. The knockout lines *osarf17-5*, *osarf17-6* and *osarf17-8* were obtained courtesy of Prof. Hongwei Xue [9], and the *osarf17-2-1*, *osarf17-5-2* knockout lines and the *OsARF17* over-expressing lines were generated previously [12]. All primers used for gene constructs are listed in Table S1. The strains and reagents are listed in Table S2. A total of 31 *U. virens* isolates used for sequence alignment of *SCRE4* are listed in Table S3 [33].

### 4.2. Plasmid Constructs and Agrobacterium-Mediated Rice Transformation

The pUC19-*35S:SCRE4-3×FLAG* construct was generated by introducing coding sequences amplified from cDNA of *U. virens* into pUC19-*35S:3×FLAG* after *Xho* I and *Bst*B I digestion. The pUC19-*35S:SCRE4^NM^-3×FLAG* was generated through site-directed mutagenesis. In order to generate SCRE4-OE lines, the CDS of *SCRE4* was amplified and subcloned into pC1305-*3×FLAG* after digestion with *Kpn* I and *Hin*d III [53]. The promoter region of *OsARF17* was amplified and subcloned into pUC19-*LUC* to construct pUC19-*ProOsARF17:LUC*. *GFP* coding sequence was amplified and ligated into the vector pUC19-*ProOsARF17:LUC* to replace the *LUC* coding sequence after *Xho* I and *Pst* I digestion.

In order to generate the transgenic lines with DEX-induced expression of SCRE4, the coding sequences of *SCRE4-3×FLAG* and *SCRE4^NM^*-*3×FLAG* were amplified and ligated into pTA7001 after *Xho* I and *Spe* I digestion [54]. Different constructs were introduced into *Agrobacterium* strain EHA105 using the freeze-thaw method [55] and were then transformed into rice cultivar Nipponbare through *Agrobacterium*-mediated transformation as described previously [56,57].

### 4.3. RNA Isolation and Quantitative RT-PCR

Total RNAs were extracted from rice seedlings and inoculated rice panicles using an Ultrapure RNA kit (CWBio, Beijing, China) following the manufacturer’s instructions and were quantified via NanoDrop 2000 (Thermo Fisher Scientific, Waltham, MA, USA). Complementary DNA was synthesized using total RNAs as the template with Superscript III reverse transcriptase (Invitrogen, Carlsbad, CA, USA). Quantitative RT-PCR (RT-qPCR) was performed with SYBR premix Ex Taq (CWBio) using an ABI PRISM 7000 Sequence Detection System (Applied Biosystems, Foster City, CA, USA) by gene-specific primers (Table S1).

### 4.4. Yeast Secretion Assay

Yeast secretion assay was performed as described previously [33,40,41]. The predicted signal peptide-coding sequence of *SCRE4* was amplified using the primers listed in Table S1 and was subcloned into pSUC2T7M13ORI (pSUC2), in which the truncated *SUC2* gene encodes invertase without signal peptide [58]. The construct was then transformed into the invertase deficient yeast strain YTK12 using a Frozen-EZ yeast transformation II kit (Zymo Research, Irvine, CA, USA). The transformants were selected on a CMD-W medium (0.67% yeast N base without amino acids, 0.075% tryptophan dropout supplement, 2% sucrose, 0.1% glucose and 2% agar) and were then cultured on a YPRRA medium (1% yeast extract, 2% peptone, 2% raffinose and antimycin A at 2–4 mg L^−1^) to determine the functionality of predicted signal peptide.

### 4.5. Transient Expression in N. benthamiana and Rice Protoplasts

For transient expression in *N. benthamiana*, overnight-cultured *Agrobacterium* strains were collected and washed twice with distilled water and were then re-suspended in induction buffer (10 mM MES, pH 5.7, 10 mM MgCl_2_ and 150 mM acetosyringone) to an optical density at 600 nm (OD_600_) of 0.5. After incubation for 3–4 h at 28 °C, *Agrobacterium* strains carrying different gene constructs were infiltrated into 4-week-old *N. benthamiana* leaves with needleless syringes.

The transfection of rice protoplasts was performed as described previously [59]. Briefly, 3-week-old rice seedlings without roots were sliced into small pieces with a surgical blade. Sliced pieces were incubated in the lyase mixture (10 mM MES, pH 5.7, 0.6 M mannitol, 1% cellulase RS, 0.5% macerozyme R10, 0.1% bovine serum albumin, 1 mM CaCl_2_, 5 mM β-mercaptoethanol) and were gently shaken at 28 °C in darkness for 4–6 h. The protoplasts were collected by low-speed centrifugation after filtering and were then washed with W5 buffer (154 mM NaCl, 125 mM CaCl_2_, 5 mM KCl, 2 mM MES, pH 5.7) twice. The collected protoplasts were gently suspended with MMG buffer (4 mM MES, pH 5.7, 0.8 M mannitol and 1 mM MgCl_2_) and were adjusted to the density of 1.5–2.5 × 10^6^ mL^−1^. After incubation in an ice bath for 30 min, aliquots of rice protoplasts (200 µL) were mixed with 220 µL of PEG buffer (40% PEG4000, 0.6 M mannitol and 1 mM CaCl_2_) and 10 µg of plasmid DNA. The mixture was incubated for 10 min at 28 °C, and then W5 buffer was added to stop transfection. The protoplasts were collected by centrifugation at 1, 200 rpm for 3 min and were re-suspended in W5 buffer. The protoplasts were kept in the dark for 12–16 h at 28 °C for protein expression.

### 4.6. Protein Extraction and Western Blotting

Total proteins were extracted from rice leaves and the transfected rice protoplasts following the described procedure [60,61]. The proteins were separated by 10% SDS-PAGE gel and were then electroblotted onto PVDF membrane (Millipore, Bedford, MA, USA). The blots were probed with different antibodies as indicated. The chemiluminescence signals were detected using Pierce ECL Western blotting substrate (Thermo Fisher Scientific). The relative protein levels were quantified by ImageJ software (v1.51k, National Institutes of Health, Bethesda, MD,USA).

### 4.7. Subcellular Localization

The CDS of *SCRE4^*Δ*SP^* was amplified and subcloned into pGD-RFP [62] using the primers listed in Table S1. The constructs were transformed into *Agrobacterium* EHA105, as described above. The transformed *Agrobacterium* strains were agro-infiltrated into *N. benthamiana* leaves to express SCRE4^ΔSP^-RFP. *N. benthamiana* leaves were collected at 48 h post infiltration (hpi) and were stained with 4, 6-diamidino-2-phenylindole (DAPI) as described previously [33]. Briefly, the detached leaves were soaked into 5 mg mL^−1^ of DAPI solution with 0.01% Silwet L-77 for 10 min and were then rinsed with distilled water three times. For subcellular localization in rice protoplasts, the CDS of *SCRE4^*Δ*SP^* was amplified and subcloned into pRTV-GFP [63]. The pRTV-*SCRE4^*Δ*SP^-GFP* and pRTV-*SCRE4^NM^-GFP* were transfected into rice protoplasts isolated from the seedlings. RFP-NLS was constructed as a nuclear localization marker. Red and green fluorescence was observed in *N. benthamiana* cells and in rice protoplasts using a Leica SP8 confocal/multiphoton microscope system (Leica Microsystems, Mannheim, Germany).

### 4.8. Southern Blot Analysis

Southern blot analysis was performed as described previously [64]. Briefly, genomic DNA was isolated from the wild-type strain and *scre4* knockout candidates using CTAB extraction buffer (100 mM Tris-Cl, pH 8.0, 1.4 M NaCl, 20 mM EDTA, 3% CTAB, 0.2% β-mercaptoethanol) and was then digested with *Pst* I overnight. The digested DNA was separated in 1% agarose gel and was then blotted on the Hybond^TM^-N^+^ membrane (GE Healthcare, Amersham, UK). The membrane was hybridized with the digoxin-labeled probe using a DIG High Prime DNA Labeling and Detection Starter Kit II (Roche, Basel, Switzerland) according to the manufacturer’s instructions.

### 4.9. Effector Translocation Assay

The CDS of *SCRE4* was amplified and subcloned into the pYF11-*ProRP27:GFP* vector via homologous recombination in *Saccharomyces cerevisiae* [65]. The plasmid was isolated from *S. cerevisiae* with a yeast plasmid extraction kit (Solarbo, Beijing, China). After amplification in *E. coli* DH5α, the plasmid was transformed into *M. oryzae* Guy11 via PEG-mediated protoplast transformation, as described previously [66]. The transformants with strong green fluorescence were selected for inoculation assays. The conidial suspension (10^5^ spores mL^−1^) was prepared and injection-inoculated into rice leaf sheaths. Green fluorescence in the BICs and nuclei was observed under confocal microscopy after the inoculated leaf sheaths were incubated in a growth chamber in darkness at 28 °C for 30 h and for 42 h, respectively.

### 4.10. Ustilaginoidea virens Inoculation Assay

*Ustilaginoidea viren* isolates were cultured in potato sucrose broth (PSB) for 5–7 days with shaking at 140 rpm at 28 °C. The cultures were smashed by a blender, and the conidial suspension was then adjusted to 1.5 × 10^6^ spores mL^−1^ for PJ52 and to 4 × 10^6^ spores mL^−1^ for JS60-2. Rice panicles were inoculated by injection, as described previously [67,68]. The hyphal and conidial mixtures (1 mL) of the wild-type P1, *scre4* mutant and complemented strains were injected into rice panicles of the susceptible rice cultivar LYP9 with a syringe at the late booting stage (about 5–7 days before heading). PSB was injected into rice panicles as mock control. The rice cultivar Nipponbare and its derivative transgenic lines were inoculated with JS60-2, while the cultivar ZH11 and its derivative transgenic lines were inoculated with PJ52. False smut balls formed on rice panicles were counted at 4 weeks after inoculation.

### 4.11. Dual-LUC Reporter Assay

The dual-LUC reporter assay was performed in *N. benthamiana* leaves as previously described [12]. The promoter of *OsARF17* was cloned into pGreenII 0800-*LUC* to drive the expression of the firefly luciferase gene (*LUC*). The expression of the Renilla luciferase gene (*REN*) under the control of the *35S* promoter was used as a reference. The *SCRE4*, *UV8b_03835* and *UV8b_03279* genes were cloned into pGD-*RFP*. The constructs were transformed into the *Agrobacterium* GV3101 strain carrying the helper plasmid pSoup. Different *Agrobacterium* strains expressing distinct effectors and reporters were agro-infiltrated into *N. benthamiana* leaves. The dual-LUC assays were performed at 72 hpi using the Dual-Lumi^TM^ Ⅱ luciferase Assay Kit (YEASEN, Shanghai, China) according to the manufacturer’s instructions. The ratio of LUC/REN represented the relative luciferase activity.

### 4.12. ROS Burst Assay

PAMP-induced ROS burst was detected as described previously [69,70]. Briefly, leaf discs collected from 6-week-old rice plants were incubated in sterile water overnight and were then treated with chitin (10 μg mL^−1^) in the reaction buffer containing 40 mM Tris-Cl, pH 7.5, 4 µM Immun-Star HRP substrate (Bio-Rad, Hercules, CA, USA), and 50 µg mL^−1^ peroxidase-streptavidin (Solarbio). Luminescence signal was detected immediately after chitin treatment by Glomax 20/20 Luminometer (Promega, Madison, WI, USA).

### 4.13. MAPK Activation Assay

MAPK activation was detected following the described procedure [35,71]. Briefly, rice seedlings were treated with chitin (10 μg mL^−1^) or sterile H_2_O with 0.01% Silwet L-77 (GE Healthcare). The treated seedlings were collected for protein extraction at 15 min after treatment. Total protein extracts were subjected to immunoblotting with anti-phospho-p44/42 MAPK antibody (Cell Signaling Technology, Danvers, MA, USA).

### 4.14. Statistical Analysis

Significance analyses were performed with one-way ANOVA followed by Duncan’s multiple range test using SPSS software (SPSS 19.0, IBM, Armonk, NY, USA). Pairwise comparisons were performed by Student’s *t*-test using Microsoft Excel.

## Data Availability

The data that support the findings of this study are available from the corresponding author upon reasonable request.

## Supplementary Materials

The supporting information can be downloaded at: https://www.mdpi.com/article/10.3390/ijms231810527/s1.
