# Supplementary material for: Ustilaginoidea virens Nuclear Effector SCRE4 Suppresses Rice Immunity via Inhibiting Expression of a Positive Immune Regulator OsARF17
Source: Int J Mol Sci. 2022 Sep 10;23(18):10527. doi: 10.3390/ijms231810527 (PMC9501289; doi:10.3390/ijms231810527)
Supplement: Supplementary file 1 [file ijms-23-10527-s001.zip › ijms-1878339-supplementary.pdf]

Supplementary Material

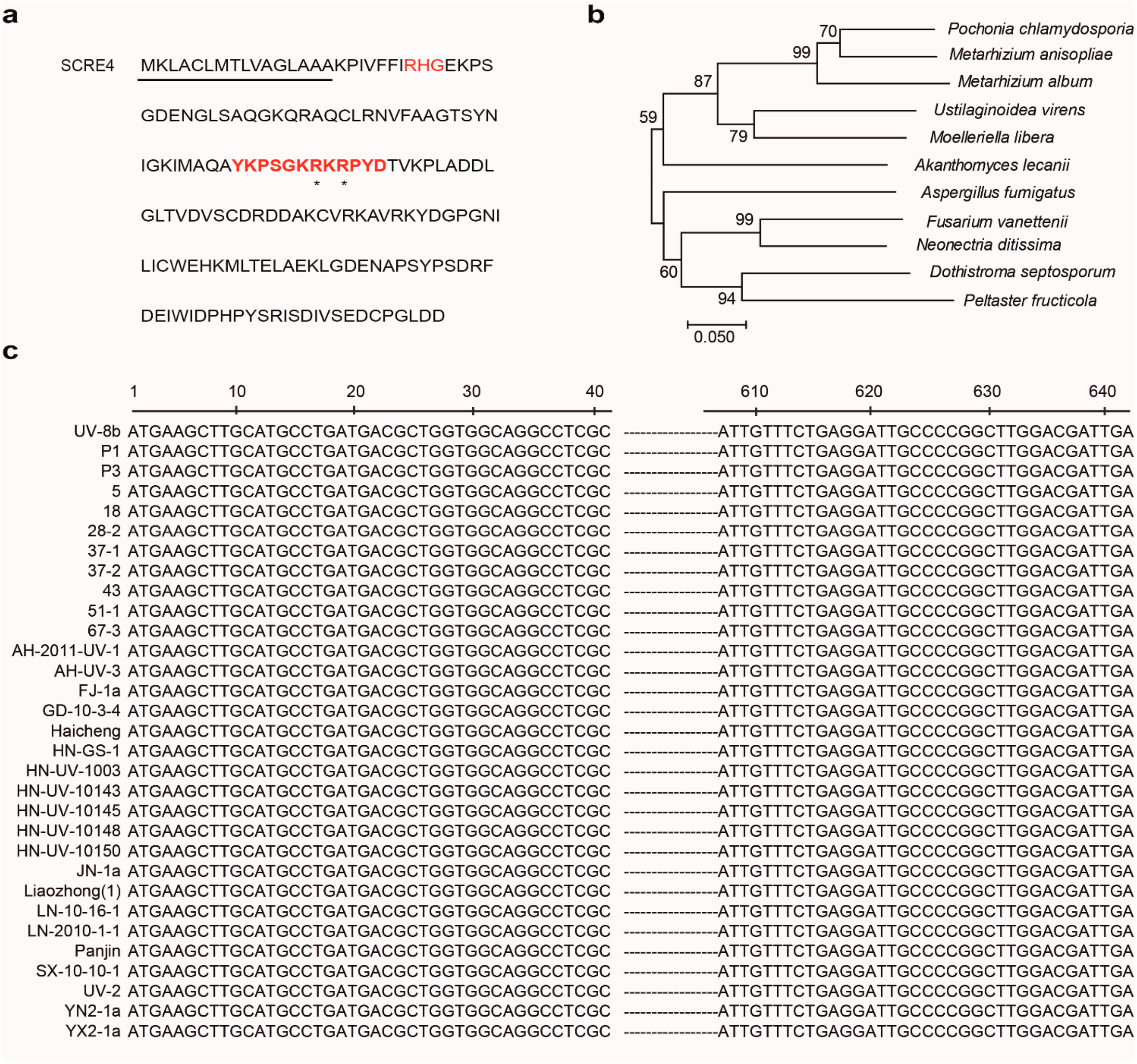

Figure S1. The highly conserved protein SCRE4 in *Ustilaginoidea virens* contains the putative signal peptide and nuclear localization signal. (**a**) The amino acid sequences of SCRE4 in *U. virens*. The signal peptide predicted by SignalP-5.0 is underlined, and the putative nuclear localization signal (NLS) predicted by cNLS mapper is highlighted in bold and red, in which the two conserved basic residues Arg73 and Arg75 are indicated by asterisks. 'RHG' motif is highlighted in red. (**b**) The phylogenetic tree generated for SCRE4 homologs from different fungal species predicted by BLASTp. The neighbor-joining method was used to construct the tree using MEGA 7.0 program. Accession numbers for the sequences of SCRE4 homologs in different fungal species are as follows: *Pochonia chlamydosporia* XP_018139175.1, *Metarhizium anisopliae* KFG79890.1, *Metarhizium album* XP_040675940.1, *Ustilaginoidea virens* XP_043001097.1, *Moelleriella libera* OAA33337.1, *Akanthomyces lecanii* OAR00280.1, *Aspergillus fumigatus* OXN06976.1, *Fusarium vanettenii* XP_003047861.1, *Neonectria ditissima* KPM34476.1, *Dothistroma septosporum* EME39468.1 and *Peltaster fructicola* QIX00063.1. (**c**) Alignment analysis of *SCRE4* coding sequences of 31 *U. virens* isolates collected from different regions in China.

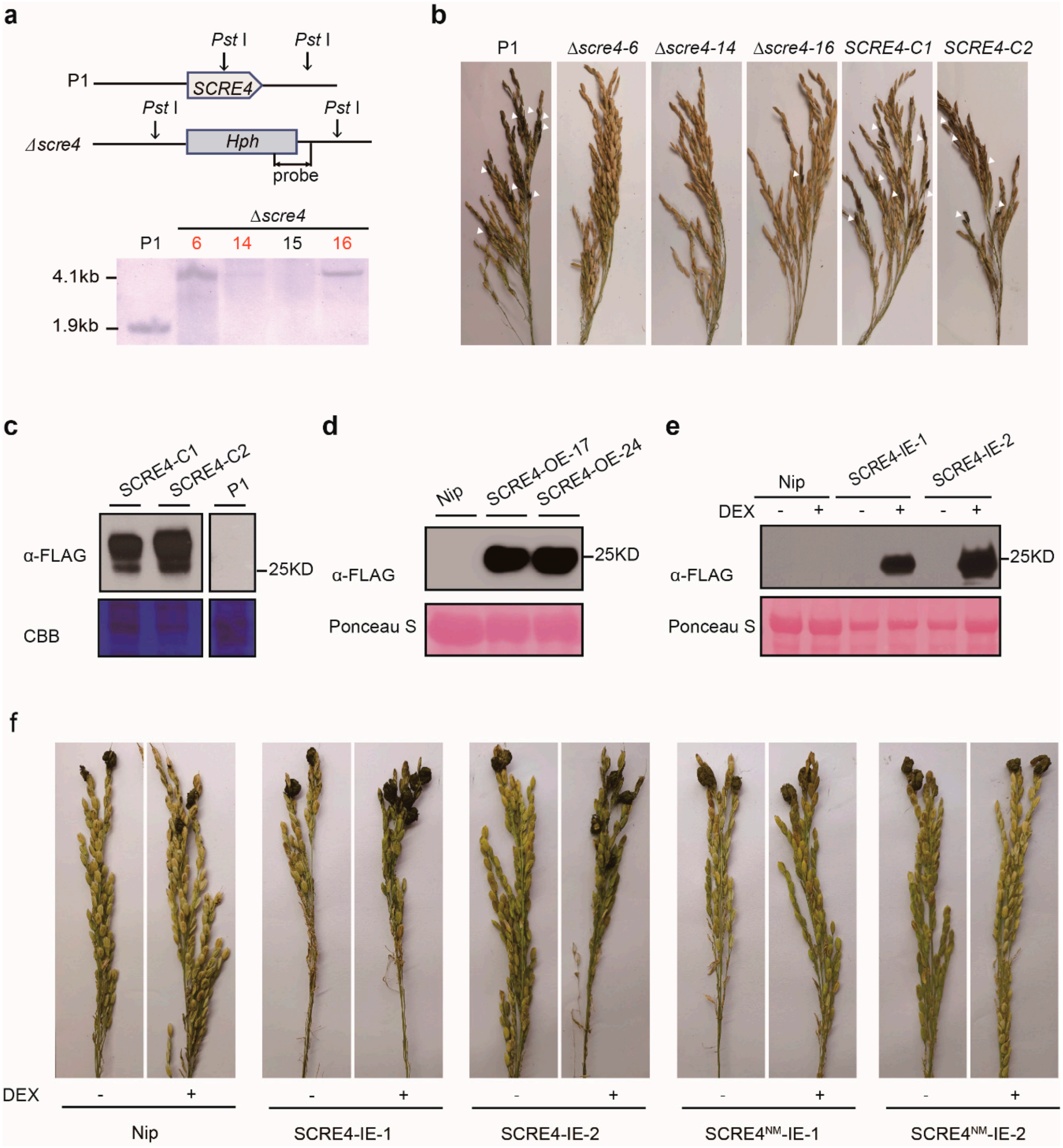

Figure S2. SCRE4 is an essential virulent factor. (**a**) Southern blot analysis to confirm the *scre4* knockout mutants. Genomic DNA isolated from the wild-type and candidate *scre4* knockout lines was digested by *Pst* I overnight. After being separated on agarose gel, the digested DNA was blotted on nylon membrane and was hybridized with the digoxin-labelled probe. The confirmed mutants were marked in red. (**b**) Disease symptoms on the representative rice panicles after *U. virens* inoculation. The images were captured at 4 weeks after rice panicles of LYP9 were inoculated by the wild-type (P1), different *scre4* knockout and complemented strains (*SCRE4-C1* and *SCRE4-C2*), the positions of false smut balls were indicated by white triangles. (**c**) Western blot analyses to detect expression of FLAG-tagged SCRE4 in complemented strains (*SCRE4-C1* and *SCRE4-C2*). Protein loading is indicated by Coomassie Brilliant Blue (CBB). (**d-e**) Western blot analyses to detect expression of FLAG-tagged proteins in the SCRE4-OE-17/-24 lines (**d**), and SCRE4-IE-1/-2 transgenic lines (**e**). Total proteins were extracted from the seedlings of the transgenic lines and were detected with an anti-FLAG (α-FLAG) antibody. The SCRE4-IE-1/-2 and SCRE4^{NM}-IE-1/-2 transgenic seedlings were treated with DEX (1 μM) and mock before protein extraction. Protein loading is indicated by Ponceau S staining. (**f**) Disease symptoms on the representative panicles of the SCRE4-IE and SCRE4^{NM}-IE transgenic lines after *U. virens* inoculation. The images were captured at 4 weeks after the transgenic lines were inoculated with the *U. virens* strain JS60-2. The SCRE4-IE and SCRE4^{NM}-IE transgenic lines were treated with DEX and mock at 24 h before *U. virens* inoculation.

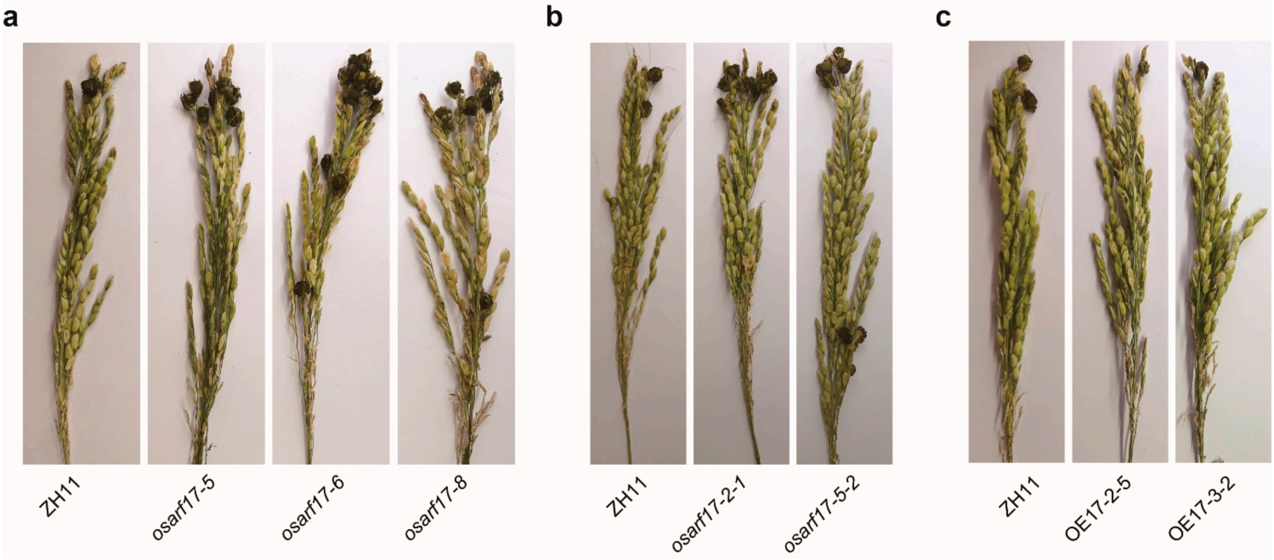

Figure S3. OsARF17 positively regulates rice resistance against *U. virens* infection. (**a-b**) Disease symptoms on inoculated panicles of the wild-type (ZH11) and different *osarf17* mutant lines. The *osarf17-5*, *osarf17-6*, and *osarf17-8* knockout lines in (**a**) and *osarf17-2-1* and *osarf17-5-2* knockout lines in (**b**) were generated with two distinct sgRNAs. (**c**) Disease symptoms on inoculated panicles of the wild-type, OE17-2-5 and OE17-3-2 transgenic lines. (**a-c**) The representative images were captured at 4 weeks after the wild-type and transgenic plants were injection inoculated with the *U. virens* isolate PJ52.

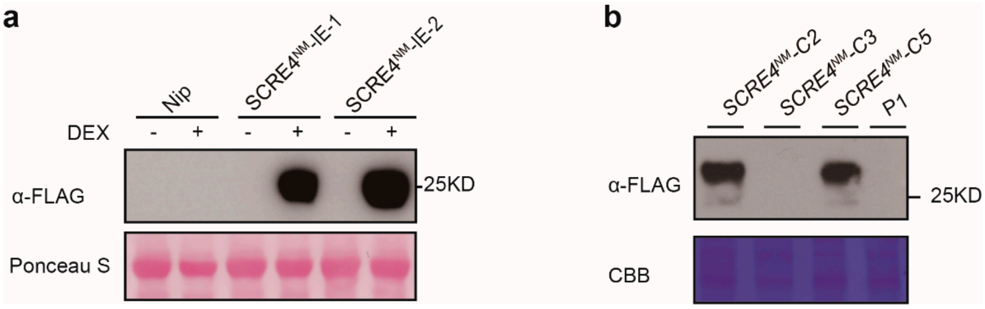

Figure S4. SCRE4^{NM}-FLAG expression was detected via immunoblotting. (**a**) Western blot analyses to detect expression of FLAG-tagged proteins in the SCRE4^{NM}-IE-1/-2 transgenic lines. Total proteins were extracted from the seedlings of the transgenic lines and were detected with an anti-FLAG (α-FLAG) antibody. SCRE4^{NM}-IE-1/-2 transgenic seedlings were treated with DEX (1 μM) and mock before protein extraction. Protein loading is indicated by Ponceau S staining. (**b**) Western blot analyses to detect expression of FLAG-tagged SCRE4^{NM} in complemented strains (*SCRE4^{NM}-C2*and *SCRE4^{NM}-C5*). Protein loading is indicated by CBB.

Table S1. The primers in this study

Primer name	sequence
**pSUC2**	
pSUC2-SCRE4-EcoRI-F	attgaattcatgaagcttgcatgcctgatgac
pSUC2-SCRE4-XhoI-R	attctcgagactcagtccgttctcgtcgc
**pYF11**	
pYF11-RP27:SCRE4-F	tttcgtaggaacccaatcttcaaaatgaagcttgcatgcctgatgac
pYF11-RP27:SCRE4-R	caccaccccggtgaacagctcctcgcccttgctcacatcgtccaagccggggcaat
**Primers for construction of *SCRE4* knockout mutant and complemented strains**	
CRISPR-SCRE4-F	acct gcttgcatgcctgatgacgc
CRISPR-SCRE4-R	aaacgcgtcatcaggcatgcaagc
SCRE4-S1-F	ggtgccgcagacaagggtac
SCRE4-S2-R	gccgaccgggaaccagttatcggcggatgtcccccgacta
SCRE4-S3-F	tagtcgggggacatccgccgataactggttcccggtcggc
SCRE4-S4-R	gcttgccccatcatccagtttggtttagtcgtcaggcggt
SCRE4-S5-F	caccgcctgacgactaaaccaaactggatgatggggcaagc
SCRE4-S6-R	ctcagccgtgcaggaaccagcaat
pY2P102-SCRE4-XhoI-F	attctcgagggcgattggcgttttcacga
pY2P102-SCRE4-EcoRI-R	attgaattcatcgtccaagccggggcaatc
Soeing-SCRE4^{NM}-F	tagtcgggggacatccgccatgaagcttgcatgcctgat
Soeing-SCRE4^{NM}-R	atcaggcatgcaagcttcatggcggatgtcccccgacta
**Primers for transient expression in *Nicotiana benthamiana***	
pGD-SCRE4$^{\Delta SP}$-RFP-XhoI-F	ctctacaagatctcgagatgaaaccaatcgtcttcttcatccgc
pGD-SCRE4$^{\Delta SP}$-RFP-SalI-R	ggaggaggccatgtcgacatcgtccaagccggggcaat
**Primers for transient expression in rice protoplast**	
pUC19-ProOsARF17:GFP-XhoI-F	attctcgagatggtgagcaagggcgagga
pUC19-ProOsARF17:GFP-PstI-R	attctgcagttacttgtacagctcgtccat
pUC19-SCRE4$^{\Delta SP}$-3×FLAG-XhoI -F	attctcgagatgaaaccaatcgtcttcttcatccgc
pUC19-SCRE4$^{\Delta SP}$-3×FLAG-BstBI-R	attttcgaaatcgtccaagccggggcaat
pRTV-SCRE4$^{\Delta SP}$-cGFP-SacI-F	ggatccccgggtgagctcatgaaaccaatcgtcttcttcatccgc
pRTV-SCRE4$^{\Delta SP}$-cGFP-HindIII-R	cgcactagtaagcttatcgtccaagccggggcaat
pRTV-SCRE4$^{\Delta SP}$-cRFP-BamHI-F	cagatccagtgggatccatgaaaccaatcgtcttcttcatccgc
pRTV-SCRE4$^{\Delta SP}$-cRFP-HindIII-R	cgcactagtaagcttatcgtccaagccggggcaat
pRTV-RFP-NLS-BamHI-F	cagatccagtgggatccatgagcgagctgatt
pRTV-RFP-NLS-Mid-R	ttatacttttctctttttctttggcttgtacagctcgtccatgccgcccttgtgcccca
pRTV-RFP-NLS-HindIII-R	cgcactagtaagcttttatacttttctctttttctttggc
pUC19-UV8b_03835$^{\Delta SP}$-3×FLAG-XhoI-F	attctcgagatgactccgtgccatgctcccga
pUC19-UV8b_03835$^{\Delta SP}$-3×FLAG-BstBI-R	atttcgaaaggtttgca aaaatccccggta
pUC19-UV8b_03279$^{\Delta SP}$-3×FLAG-XhoI -F	attctcgagatgatccctcgaacgccacccat
pUC19-UV8b_03279$^{\Delta SP}$-3×FLAG-BstBI-R	attttcgaagggcgaatctgtatcaatatctgt
**Primers for construction of transgenic rice plants**	
pTA7001-SCRE4-XhoI-F1	attctcgagatgaagcttgcatgcctgatgac
pTA7001-SCRE4^{NM}-R2	cggggcctttgccttgccacttggcttgtaggcctgggcc
pTA7001-SCRE4^{NM}-F3	gtggcaaggcaaaggccccgtacgatactgtcaagccgct
pTA7001-SCRE4-3FLAG-SpeI-R4	attactagttcacttatcgtcatcg
pC1305-SCRE4$^{\Delta SP}$-FLAG-KpnI-F	gttgtacagagctcggtaccatgaaaccaatcgtcttcttcatccgc
pC1305-SCRE4$^{\Delta SP}$-FLAG-HindIII-R	tggtctttgtagtcaagcttatcgtccaagccggggcaat
**Primers for Dual-LUC reporter assays**	

pGreenII0800-proOsARF17-KpnI-F	ctcactatagggcgaattgggtacctctaaattataatccac
pGreenII0800-proOsARF17-PstI-R	aactagtggatcccccgggctgcagttttcttctctttctctctctctc
pGD-UV8b_03835$^{\Delta SP}$-RFP-XhoI-F	ctctacaagatctcgagatgatccctcgaacgccacccat
pGD-UV8b_03835$^{\Delta SP}$-RFP-SalI-R	ggaggaggccatgtcgacaggtttgcaaaaatccccggta
pGD-UV8b_03279$^{\Delta SP}$-RFP-XhoI-F	ctctacaagatctcgagatgatccctcgaacgccacccat
pGD-UV8b_03279$^{\Delta SP}$-RFP-SalI-R	ggaggaggccatgtcgacgggcgaatctgtatcaatatct
pGD-SCRE4$^{\Delta SP}$-RFP-XhoI-F	ctctacaagatctcgagatgaaaccaatcgtcttcttcatccgc
pGD-SCRE4$^{\Delta SP}$-RFP-SalI-R	ggaggaggccatgtcgacatcgtccaagccggggcaat
**Conservation analysis of SCRE4**	
SCRE4-F	catggagcaccgaggcactaa
SCRE4-R	gtgcagttccacgcgcaggc
**SCRE4 specific probe for Southern blot**	
SCRE4-probe-F	gcacgagattcttcgccctc
SCRE4-probe-R	tcagggcttgtttccgacga
**qRT**	
OsGAPDH-qF	aagccagcatcctatgatcagatt
OsGAPDH-qR	cgtaacccagaatacccttgagttt
OsARF17-qF	cgccggaagctgtagaagaa
OsARF17-qR	agctacctgttcgctgtgac
SCRE4-qF	gcacaagatgctcacggaac
SCRE4-qR	cggggcaatcctcagaaaca
α-tubulin-1-qF	aggttgcgttgaaggaggtt
α-tubulin-1-qR	gaggtggagttgccgataaa

Table S2. The strains and reagent in this study

**Strains**	**Source/ reference**
Escherichia coli DH5α	Lab collection
Agrobacterium tumefaciens	
EHA105	Lab collection
GV3101(pSoup)	ZomanBio, ZC1406
Magnaporthe oryzae	
Guy11	Zhengguang Zhang at Nanjing Agricultural University
Ustilaginoidea virens	
P1	Lab collection
JS60-2	Zhaoxi Luo at Huazhong Agricultural University
PJ52	Jing Fan at Sichuan Agricultural University
**Yeast**	
YTK12	Zymo Research
XK125	Zhengguang Zhang at Nanjing Agricultural University
**Reagent**	**Source/ reference**
anti-FLAG M2 monoclonal antibody	Sigma-Aldrich, F1804
HRP-conjugated anti-HA monoclonal antibody	Roche, 11667475001
Phospho-p44/42 MAPK (Erk1/2) (Thr202/Tyr204) Antibody	Cell Signaling Technology, 9101
anti-β-Actin Mouse monoclonal antibody	CWBIO, CW0096M
anti-GFP Mouse monoclonal antibody	LABLEAD, G1002
Goat anti-Mouse IgG, HRP Conjugated	CWBIO, CW0102S
Goat anti-Rabbit IgG, HRP Conjugated	CWBIO, CW0103S
Dual-Lumi™ II luciferase Assay Kit	YEASEN, 11402ES60

Table S3. 31 *U. virens* isolates from different regions in China

**Name**	**origin region**
Uv-8b	Huazhong Agricultural University
P1	Jiangsu Academy of Agricultural Sciences
P3	Unknown
5	Xuzhou City, Jiangsu Province
18	Xuzhou City, Jiangsu Province
28-2	Suzhou City, Jiangsu Province
37-1	Nanjing City, Jiangsu Province
37-2	Lishui County, Nanjing City, Jiangsu Province
43	Nanjing City, Jiangsu Province
51-1	Yancheng City, Jiangsu Province
67-3	Nantong City, Jiangsu Province
AH-2011-UV-1	Anhui Province
AH-UV-3	Anhui Province
FJ-1a	Nanjing County, Zhangzhou City, Fujian Province
GD-10-3-4	Guangdong Province
Haicheng	Haicheng City, Liaoning Province
HN-GS-1	Luoshan County, Xinyang City, Henan Province
HN-UV-1003	Taojiang County, Yiyang City, Hunan Province
HN-UV-10143	Hanshou County, Changde City, Hunan Province
HN-UV10145	Changde City, Hunan Province
HN_UV-10148	Xiangtan City， Hunan Province
HN-UV-10150	Anren County, Chenzhou City, Hunan Province
JN-1a	Licheng District, Jinan City, Shandong Province
Liaozhong (1)	Liaozhong District, Shenyang City, Liaoning Province
LN-10-16-1	Donggang City, Liaoning Province
LN-2010-1-1	Gushan Town, Donggang City, Liaoning Province
Panjin	Panjin City, Liaoning Province
SX-10-10-1	Nanzheng County, Hanzhong City, Shanxi Province
UV-2	Jiangshu Academy of Agricultural Sciences
YN2-1a	Yunnan Province
YX2-1a	Yangxin County, Huangshi City, Hubei Province
